# Enhancing Digestibility and Intestinal Peptide Release of *Pleurotus eryngii* Protein: An Enzymatic Approach

**DOI:** 10.3390/jof10120890

**Published:** 2024-12-23

**Authors:** Dandan Wang, Meng Zhang, Jianing Wan, Haiquan Liu, Ying Wang, Ruiheng Yang, Yingying Wu, Dapeng Bao, Hongyu Chen, Gen Zou, Yong Zhao

**Affiliations:** 1 College of Food Sciences and Technology, Shanghai Ocean University, Shanghai 201306, China; 2 National Engineering Research Center of Edible Fungi, Key Laboratory of Applied Mycological Resources and Utilization, Ministry of Agriculture, Institute of Edible Fungi, Shanghai Academy of Agricultural Sciences, Shanghai 201403, China

**Keywords:** *Pleurotus eryngii*, fungal protein, papain, neutral protease, alkaline protease, gastrointestinal digestion

## Abstract

*Pleurotus eryngii* is a tasty and low-calorie mushroom containing abundant high-quality protein. This study aims to improve the digestibility of *P. eryngii* protein (PEP) and hence to facilitate its development as a healthy alternative protein. The extracted PEP was pretreated with 1000–5000 U of papain, neutral protease and alkaline protease. The Chyme collected from in vitro simulated gastrointestinal digestion was analyzed by fluorescence microscopy and protein particle analyzer, and the endpoint profiles of peptides and amino acids were determined by UHPLC-MS/MS and NanoLC-MS/MS. The particle size curve and fluorescence microscopy images jointly supported that protease hydrolysis improved decomposition and dispersion of PEP during digestion, particularly in the gastric phase. The impact on Zeta potential was minimal. Proteases effectively increased the abundance of amino acids after digestion, particularly L-isomer Lys and Arg Maximum release was achieved when pretreated with 5000 U of alkaline protease, reaching 7.54 times that of control. Pretreatments by proteases also notably increased digestive yields of 16,736–19,870 peptides, with the maximum reaching 1.70 times that of the control, which mainly consisted of small peptides composed of 7–15 amino acids with molecular weight below 800 Da. The findings indicated that protease hydrolysis, especially pretreatment with 5000 U of alkaline protease, effectively enhanced the digestibility of PEP, which shed light on providing enzymatic approaches for improving bioavailability and developing healthy fungal proteins.

## 1. Introduction

*Pleurotus* mushrooms rank as the second most cultivated edible fungi worldwide, accounting for 27% of the global yield [1]. *P. eryngii*, also known as king oyster or trumpet royale, is a tetrapolar heterothallic mushroom classified into the *Pleurotus* genus of basidiomycetes. It is nutritious, chewy and low in calories, and therefore continuously popular among consumers across the world. Many studies have demonstrated that *P. eryngii* has positive impacts on human health, such as anti-bacterial, anti-inflammatory and anti-oxidation effects, gut microbiota improvement, and immune regulation [2,3,4]. Therefore, it has great potential in the development of green, healthy, and sustainable foods. In recent years, the *P. eryngii* industry has grown rapidly. According to statistics from the China Edible Fungi Association, the yield of *P. eryngii* reached 2.0518 million tons in 2021, ranking second among factory-cultivated edible mushrooms in China, accounting for 22.10% of the total production [5].

In the past few decades, there has been growing interest in seeking healthy and eco-friendly protein alternatives. Dietary proteins are found in a variety of sources. The traditional meat and poultry products, such as chopped beef, pork and chicken, contain protein ranging from 47 to 71% DW (on a dry weight basis). Legumes, such as peanuts, soybeans, chickpeas and tofu, have protein contents between 23 and 52% DW. Cereal grains, such as barley, buckwheat, and wheat flour, provide 8–14% DW of protein. Vegetables typically contain 7–13% DW of protein [6] (https://fdc.nal.usda.gov (accessed on 26 November 2024)). The protein content of fungal biomass may reach up to 45% DW [7]. As a sustainable option, fungal protein is increasingly favored in the market due to its production being unaffected by animal diseases, requiring less land, and minimally impacting aquatic and atmospheric environments. *P. eryngii* protein comprises abundant and balanced amino acids [8], making it a high-quality fungal protein that can achieve year-round stable production [3]. It has an umami taste and has been reported to show versatile bioactive properties, such as immune regulation [4,9], anti-oxidation [3,10], and anti-inflammation [11]. The indispensable amino acid (IAA) content in *P. eryngii* protein reaches 589.55 mg/g pro protein, accounting for 40% of the total amino acid pool, which is higher than most plant proteins, making it a rich source of IAA [8,12,13]. Rich in lysine, *P. eryngii* protein is a preferred vegetarian alternative that compensates for the lysine deficiency often found in cereal proteins [14,15]. YANG et al. [14] reported that, among different drying methods, freeze-drying technology was an effective method to preserve the amino acids of *P. eryngii* protein from loss. In addition to its high nutritional value, *P. eryngii* protein also shows great processing properties. Our previous studies revealed that *P. eryngii* protein exhibits excellent emulsifying, foaming, water-holding, and oil-holding properties, making it highly stable in food processing and ideal for use in protein snacks, baked goods, and meat substitutes [16]. LV et al. [12] used 3D printing technology to prepare *P. eryngii* protein into a new type of bakery product with the desired fluidity and viscosity, and the addition of *P. eryngii* protein also enhanced the amino acid abundance and nutritional value of the product. In papery foods made from *P. eryngii*, protein is the predominant component, providing the product with good palatability, better nutrient density, and health benefits [17]. Nevertheless, although *P. eryngii* protein is reported to have high nutritional value and enormous processing potential, there is little research on its bioavailability.

One of the key focuses of protein nutritional assessment is bioavailability [18], with digestibility being particularly important as it directly affects the extent to which proteins can be degraded and utilized by the human body [19]. Currently, the evaluation of edible mushroom protein mainly concentrates on applying instrumental analysis to investigate the chemical composition of amino acids, yet few research works have systematically studied the mechanisms of their digestibility and absorbability [13]. Compared with animal proteins, edible mushroom proteins have lower digestibility, which is related to amino acid sequences, protein structure, cell wall composition and anti-nutritional phytochemicals [13]. Improving protein digestibility is beneficial for enhancing the bioavailability of amino acids and small peptides [20]. Pretreatment processing improves the digestibility of proteins, such as breaking the cell wall by ultrasonic and high temperature to promote protein release and degradation, as well as removing or chelating anti-nutritional phytochemicals by chemical reagents [21]. Enzymatic hydrolysis is a well-accepted method for enhancing protein digestibility [22,23], which displays the advantages of mild reaction conditions, high safety, convenient operation, and low cost [24]. Proteases are highly efficient tool enzymes specializing in targeted degradation of proteins. Their function is to cleave peptide bonds in proteins, thereby releasing smaller peptides and more amino acids [25], which enables smoother absorption into the bloodstream to promote growth, maintain health and exert physiological functions, such as anti-hypertension, anti-thrombosis and anti-oxidation effects, and satiety [26]. Currently, there are very few reports on the use of proteases to improve the bioavailability of fungal proteins.

This study aims to investigate the effects of different doses of three proteases on the in vitro digestibility of *P. eryngii* protein. The particle size curve and fluorescence microscopic morphology during simulated gastrointestinal digestion were compared. The endpoint chyme was collected, and the profiles of amino acids and peptides were determined by LC-MS/MS. Combining the analysis results, an enzymatic approach for preparing *P. eryngii* protein with higher digestibility is provided. This study sheds light on the processing and development of healthy and nutritious fungal protein substitutes.

## 2. Materials and Methods

### 2.1. Materials

#### 2.1.1. *Pleurotus eryngii*

*P. eryngii* was purchased from Qianlu Agro-Product Co., Ltd. (Shanghai, China), whose fruiting bodies are produced using its own strain and cultivation process. Fresh *P. eryngii* fruiting bodies without external damage were sliced and dried in an oven at 40 °C for 72 h. The dried slices were ground by a grinder for later use.

#### 2.1.2. Chemicals and Reagents

The chemicals and reagents used in protein extraction were of analytical grade, the reagents used in instrumental analysis were of LC-MS grade. Papain, neutral protease and alkaline protease were purchased from Yuanye (Shanghai, China). Pepsin was purchased from Macklin (Shanghai, China). Trypsin was purchased from Sangon (Shanghai, China). BCA protein quantification kit was purchased from Beyotime (Shanghai, China). Simulated fluid electrolyte solutions for gastric and intestinal phases were purchased from Coolaber (Beijing, China). Bile salts and fluorescein isothiocyanate isomer I were purchased from Sigma-Aldrich (St. Louis, MO, USA).

### 2.2. Methods

#### 2.2.1. Extraction of *P. eryngii* Protein

*P. eryngii* protein was prepared as described by Chen et al. [16] and Wu et al. [3]. The dried *P. eryngii* powder was mixed with DI water at a ratio of 1 g:40 mL. The pH of the mixture was adjusted to 11 by adding 1 M NaOH. Then, the tube was immersed in a water bath at 40 °C. After 40 min, the tube was taken out and centrifuged at 1467× *g* for 10 min to collect the supernatant, and 6 M HCl was added to adjust the pH to the protein isoelectric point. After being kept at 4 °C overnight, the tube was centrifuged at 1467× *g* for 15 min. The precipitate was collected and washed with DI water for three times. The *P. eryngii* protein powder was obtained by freeze-drying (CK1).

#### 2.2.2. Enzymatic Lysis Process

Papain, neutral protease and alkaline protease were tested for hydrolysis efficiency and optimized doses. The prepared *P. eryngii* protein was added with 1000–5000 U of the above proteases and mixed with water at a ratio of 1 g:5 mL. The enzymatic lysis process was carried out according to the manufacturer’s instructions and references [27]. The reaction conditions were set as follows: 90 min, 40 °C, pH 7 (for papain and neutral proteases) or pH 10 (for alkaline protease) (Table 1). After lysis, the reaction was stopped by immersing the tube in a boiling water bath for 10 min. The mixture was centrifuged at 1467× *g* for 15 min, and then the precipitate was collected and freeze-dried. Then, the protease-pretreated *P. eryngii* proteins were harvested. On account of the potential impact of the alkaline environment on the results, an additional control was prepared by parallelly processing *P. eryngii* protein under pH 10 without adding proteases (CK2).

The yield rate was calculated as follows:

(1) $$yield\;rate=\frac{weight\;of\;protease\;treated\;protein\;(g)\;}{weight\;of\;crude\;protein\;(g)}\times 100\%\;$$

#### 2.2.3. Determination of Soluble Protein Concentration

Soluble protein concentration was measured using a commercially available BCA protein quantification kit (Beyotime, Shanghai, China) following the manufacturer’s directions. Thus, 10 mg of the samples was dissolved in 10 mL PBS to prepare the solution at 1 mg/mL. Then, 20 μL of standards (BSA) and the prepared sample solutions were incubated with 200 μL of working solutions in a 96-well microplate at 37 °C for 30 min, and then the absorbance was measured at 562 nm by TECAN Infinite200 PRO (Tecan AG, Männedorf, Switzerland). The protein concentrations of the samples were quantified by referring to the standard curve. The soluble protein content was calculated as follows.

(2) $$Soluble\;protein\;content\;(\%)=\frac{concentration\;of\;soluble\;protein\;(mg/mL)\;}{concentration\;of\;sample\;solution\;(mg/mL)}\times 100\%$$

#### 2.2.4. In Vitro Digestion

In vitro digestion was conducted using INFOGEST model as described in previous studies [28,29]. The selected samples (based on Section 2.2.2.) and reference controls were mixed with water at 1 g:3 mL and placed into a 50 mL Falcon tube.

Gastric phase: the prepared samples and controls were added with simulated gastric juice at a volume ratio of 1:1. The concentration of pepsin in the reaction system was 2000 U/mL. The tube was immersed in a water bath at 37 °C and the pH was maintained at 3.0 throughout the process. At 0 min and 180 min of the gastric phase, 2 mL of chyme was sampled and marked as G0 and G3, respectively. The samples were immediately adjusted to pH 7 with 1 M NaOH and placed in a boiling water bath for 10 min to inactivate the enzymes. Then, the samples were subsequently cooled on ice and stored at −80 °C for later use.

Intestinal phase: the chyme collected after the gastric phase was added with simulated intestinal fluid at a volume ratio of 1:1. The systematic concentration of trypsin was 100 U/mL, and the bile salt concentration was 10 mM. The tube was immersed in a water bath at 37 °C and the pH was maintained at 7.0 throughout the process. At the 0 min and 120 min of the intestinal phase, 2 mL of samples was taken and marked as I0 and I2, respectively. The samples were immediately placed in a boiling water bath for 10 min to inactivate the digestive enzymes. Then, the samples were cooled on ice and stored at −80 °C for later use.

#### 2.2.5. Particle Size Analysis

The samples collected at different digestive time points (G0, G3, I0, I2) were diluted 500 times, maintained on ice and transferred into colorimetric dishes before use. A Malvern Zetasizer analyzer (Malvern Panalytical Ltd., Malvern, UK) was preheated for 30 min and selected for size mode. The particle size of all samples was measured in triplicate.

#### 2.2.6. Zeta Potential Analysis

The samples collected at different digestive time points (G0, G3, I0, I2) were diluted 500 times, maintained on ice and transferred into colorimetric dishes before use. A Malvern Zetasizer analyzer (Malvern Panalytical Ltd., Malvern, UK) was preheated for 30 min and selected for potential mode. The zeta potential of all samples was measured in triplicate.

#### 2.2.7. Fluorescence Microscopy

The samples collected at different time points were stained and observed using a Zeiss Colibri 7 fluorescence microscope (Zeiss Group, Gottingen, Germany) under ultraviolet excitation mode. Then, 10 mg of fluorescein isothiocyanate (FITC) isomer I was dissolved in 1 mL of ultrapure water and then mixed with the sample at volume ratio of 1:1 for staining. A drop of the mixture was added to the center of a glass slide and observed in the bright field under an LD A-Plan 5×|0.15 M27 objective lens. Micrographs were taken under UV using an Axiocam 512 color digital camera (Zeiss Group, Gottingen, Germany) and observed using the accompanying Zeiss Observer 3 device.

#### 2.2.8. UHPLC-MS/MS Analysis for Amino Acid Composition

The endpoint digestive products (I2) were analyzed for amino acid composition by UHPLC-MS/MS. The samples were thawed on an ice water bath and vortexed for 30 sec to mix well. Then, 50 μL of the sample was transferred into a 1.5 mL EP tube and added with 200 μL of precooled (at −40 °C) extraction solution (acetonitrile/methanol at a volume ratio of 1:1, containing isotopic internal standard mixture). The mixture was vortexed for 30 s and sonicated in an ice ultrasonic bath for 15 min. The mixture was placed at −40 °C for 1 h and then centrifuged at 4 °C, 13,800× *g*, for 15 min. Then, 100 μL of supernatant was evaporated to dryness and then dissolved in 100 μL of 50% methanol aqueous solution. The mixture was added with 100 μL of derivatizing agent and 50 μL of 1 M NaHCO_3_, and vortexed to mix well. The derivative reaction was carried out in a water bath at 40 °C for 1 h. Then, the samples were cooled to room temperature and added with 50 μL of 2 M HCl. The mixture was evaporated to dryness and then redissolved in 200 μL of methanol before instrumental analysis.

The UHPLC was conducted using an Agilent 1290 Infinity II series UHPLC System (Agilent, Santa Clara, CA, USA), which was equipped with an ACQUITY UPLC BEH Amide column (100 × 2.1 mm, 1.7 μm, Waters, Mildord, MA, USA). The chromatographic conditions were set as follows: mobile phase A: 5 mM ammonium acetate; mobile phase B: acetonitrile; column temperature: 45 °C, auto-sampler disk temperature: 4 °C, injection volume: 2 μL.

The Thermo Altis TSQ Plus Mass Spectrometer (Thermo Fisher, Waltham, MA, USA) equipped with an ESI electric spray ion source was used for mass spectrometry. The multiple reaction monitoring (MRM) mode was selected. The ion source conditions were set as follows: spray voltage: −3300 V, sheath gas: 40 Arb, aux gas: 10 Arb, sweep gas: 1 Arb, ion transfer tube temperature: 325 °C, vaporizer temperature: 350 °C.

The quantitative analysis of target compounds was conducted using Skyline (v24.1, MacCoss Lab Software, Seattle, WA, USA), and all data collection was performed using Xcalibur (v4.4.16.14, Thermo Fisher, Waltham, MA, USA).

#### 2.2.9. NanoLC-MS/MS for Peptide Analysis

The endpoint digestive products (I2) were analyzed for peptide composition by NanoLC-MS/MS. The samples were centrifuged at 4 °C, 13,800× *g*, for 10 min. Then, 100 μL of supernatant was added with 50 μL of 2% trifluoroacetic acid (TFA). The mixture was centrifuged at 4 °C, 13,800× *g*, for 10 min. The supernatant was desalinated using a desalination column according to standard procedures, and then concentrated using a vacuum-freeze centrifuge concentrator. The samples were dissolved in mobile phase A before instrumental analysis.

The nano-flow LC was conducted using an Evosep One high-throughput LC (Evosep Biosystems, Odense, Denmark) equipped with a nano-electrospray ion source. A reversed-phase column (PePSep C18, 1.9 um, 150 um × 15 cm, Bruker GmbH, Mannheim, Germany) was applied and chromatographic conditions were set as follows: phase A: water with 0.1% (*v*/*v*) formic acid; phase B: Acetonitrile with 0.1% (*v*/*v*) FA; procedure: 44 min gradient.

Mass spectrometry was conducted using a coupled timsTOF Pro2 spectrometer (Bruker GmbH, Mannheim, Germany). The DDA PaSEF mode was selected for DDA data acquisition, and the conditions were set as follows: scanning range: 100 to 1700 *m*/*z* for MS1, linear impact energy increase pattern with ion mobility: 20 eV (1/K0—0.6 Vs/cm^2^) to 59 eV (1/K0 = 1.6 Vs/cm^2^).

#### 2.2.10. Statistical Analysis

The results were expressed as mean ± SD. Histograms, diagrams and heat maps were drawn using Origin (version 2021, Origin Lab, Northampton, MA, USA). One-way analysis of variance (ANOVA) analysis combined with post hoc Duncan Test was conducted to compare the statistical differences (*p* < 0.05) among the sample groups.

## 3. Results

### 3.1. Effect on Yield and Soluble Portion of Hydrolyzed Protein

**Figure 1:**
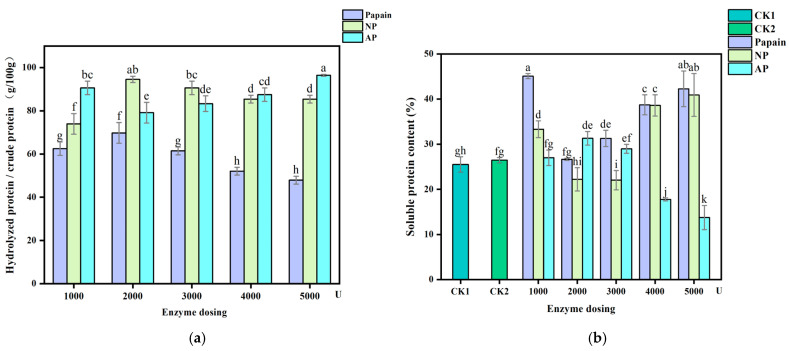
The effect of protease pretreatment on yield and soluble portion of hydrolyzed protein harvest. (**a**) Yield rate of hydrolyzed protein from crude *P. eryngii* protein. (**b**) Soluble protein content (%) of hydrolyzed protein. CK1: crude *P. eryngii* protein powder; CK2: *P. eryngii* protein parallelly processed under pH 10 without adding proteases; Papain: papain-pretreated group; NP: neutral-protease-pretreated group; AP: alkaline-protease-pretreated group. Variance among treatment groups was compared by one-way analysis of variance (ANOVA) and post hoc Duncan Test. Groups sharing no common letter are significantly different (*p* < 0.05).

The weight ratio of harvested hydrolyzed protein to crude *P. eryngii* protein was measured to determine the impact of protease type and dose on yield (Figure 1a). Within the testing range, the neutral protease and alkaline protease groups harvested more hydrolyzed protein than the papain group when treated at the same dose (*p* < 0.05), showing less processing loss. Among the different doses of neutral protease and papain, the dose of 2000 U performed better, with yield rates of 94.58% and 69.79%, respectively. The product yield rate peaked when treated with 5000 U of alkaline protease, reaching 96.46%. The effect of enzyme action on yield is a synergic influence of both enzymatic and environmental factors such as enzyme type, diffusion capacity, substrate binding ability and pH [30,31]. For papain and neutral protease, the yield reached its maximum at a dose of 2000 U and declined even with subsequent dose increases. The reason may be attributed to the accumulation of solids in the system, which weakens the dispersion of protein powder and reduces the diffusion of enzymes. Yet, for alkaline protease, the yield continued to further increase in the dose from 2000 to 5000 U, which may be due to the fact that this enzyme acts in an alkaline environment with pH 10, and the addition of dosage provides more active catalytic sites, thereby improving enzymatic efficiency and producing more hydrolyzed proteins [32].

The soluble proportion of the harvested protein was measured by a BCA assay kit, and the results were compared with those of blank control CK1 and alkaline treatment control CK2 (Figure 1b). The soluble protein contents were determined to be 25.51% and 26.46% in CK1 and CK2, respectively. Papain pretreatment increased the soluble proportion to 26.65–45.08%, higher than that of both controls, where 1000 U and 5000 U doses showed a premier performance. When pretreated with neutral protease, three doses resulted in more soluble protein than both controls, reaching 33.32% (1000 U), 38.60% (4000 U), and 40.90% (5000 U). The soluble proportion determined in the alkaline protease group was not particularly abundant, and the highest was 31.32% when treated with 2000 U. Considering both the yield and soluble proportion, 2000 U and 5000 U were selected as the doses for subsequent experiments to further test the potential of proteases for enhancing the digestibility of *P. eryngii* protein.

### 3.2. Effect on Particle Size and Potential During Gastrointestinal Digestion

**Figure 2:**
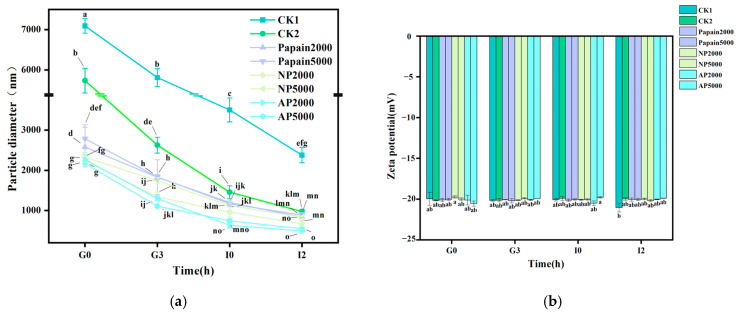
Effect of protease pretreatment on particle size (**a**) and potential (**b**) during gastrointestinal digestion. CK1: crude *P. eryngii* protein powder; CK2: *P. eryngii* protein parallelly processed under pH 10 without adding proteases; Papain: papain-pretreated group; NP: neutral-protease-pretreated group; AP: alkaline-protease-pretreated group. Variance among treatment groups was compared by one-way analysis of variance (ANOVA) and post hoc Duncan Test. Groups sharing no common letter are significantly different (*p* < 0.05).

During the in vitro simulation experiment from G0 to I2, a significant decrease in particle size was observed as the gastrointestinal digestion progressed (*p* < 0.05) (Figure 2a), suggesting the proteins were digested and broken down into smaller components. In the gastric phase, there was a statistically significant difference between both controls and the protease groups (*p* < 0.05), indicating that enzymatic hydrolysis was effective in reducing the particle size of *P. eryngii* protein. With the prolongation of digestion and the action of intestinal enzymes, the particle size of CK2 and protease groups gradually became similar, yet still shrunk more than twice compared with that of CK1. For the same protease, the influence of different doses on particle size was minimal. Among all proteases tested, the alkaline protease was the most effective in the digestion of *P. eryngii* protein into smaller fragments.

Zeta potential is an important indicator for measuring the stability of dispersed systems, as the charged groups on the surface are powerful electrostatic barriers inhibiting protein molecules from approaching and aggregating. The Zeta potential of protein chyme in both the controls and the treatment groups was negative, indicating that there were more negatively charged amino acids than positively charged ones (Figure 2b). In the intestinal phase, this phenomenon may also be attributed to the presence of anionic bile salts and peptides [33]. The difference between groups was not significant, indicating that protease has minimal impact on the potential of *P. eryngii* protein during digestion. However, the absolute potential of all groups in this study exceeded 20 mV. The absolute value of the Zeta potential is positively correlated with the number of charges and repulsion; therefore, a high absolute potential indicates that proteins are less likely to aggregate and the system tends to be stable [34]. These results indicated that the suspension systems of all groups were stable.

### 3.3. Observation Under Fluorescence Microscopy

**Figure 3:**
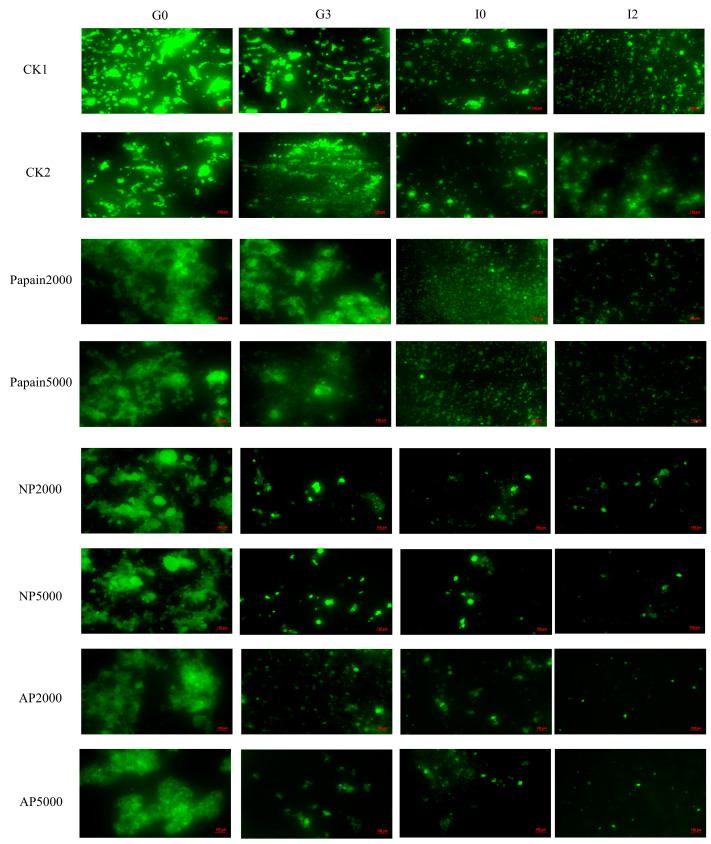
Fluorescence microscopic images collected during simulated digestion. CK1: crude *P. eryngii* protein powder; CK2: *P. eryngii* protein parallelly processed under pH 10 without adding proteases; Papain: papain-pretreated group; NP: neutral-protease-pretreated group; AP: alkaline-protease-pretreated group. Variance among treatment groups was compared by one-way analysis of variance (ANOVA) and post hoc Duncan Test.

Fluorescence microscopy was used to provide a better understanding of the spatial distribution of chyme during simulated gastrointestinal digestion, with FITC highlighting the microscopic morphology of the proteins (Figure 3). FITC isomer I is the most widely used green fluorescent derivative, characterized by excellent absorbability, fluorescence quantum yield and water solubility. Its isothiocyanate group labels the amino terminus or primary amine of proteins.

In the gastric phase, the stained proteins appeared denser and more clustered, while in the intestinal phase, collected fluorescence signals notably decreased, dispersed and became smaller, indicating that the proteins were effectively digested into small molecules. These results suggested that the intestinal phase was a crucial step in the digestion of *P. eryngii* protein, similar to Colosimo’s report on mycoprotein produced by Marlow Foods [35]. The images of CK2 and CK1 were close, indicating that the alkaline environment did not have a significant impact on the microscopic morphology of *P. eryngii* protein. The chyme in three protease-pretreated groups was digested much more thoroughly and faster than that in CKs, with smaller and more dispersed particles after the gastric phase. The proteins in the papain-pretreated and alkaline-protease-pretreated groups were more evenly distributed and less clustered at G0, while the neutral-protease-pretreated and alkaline-protease-pretreated groups showed better digestion efficiency after gastric digestion. When comparing fluorescence micrographs, all protease pretreatment groups demonstrated excellent performance, and barely any difference was observed between doses, which was consistent with the particle size analysis results.

### 3.4. Release of Amino Acids Determined by UHPLC-MS/MS Analysis

**Figure 4:**
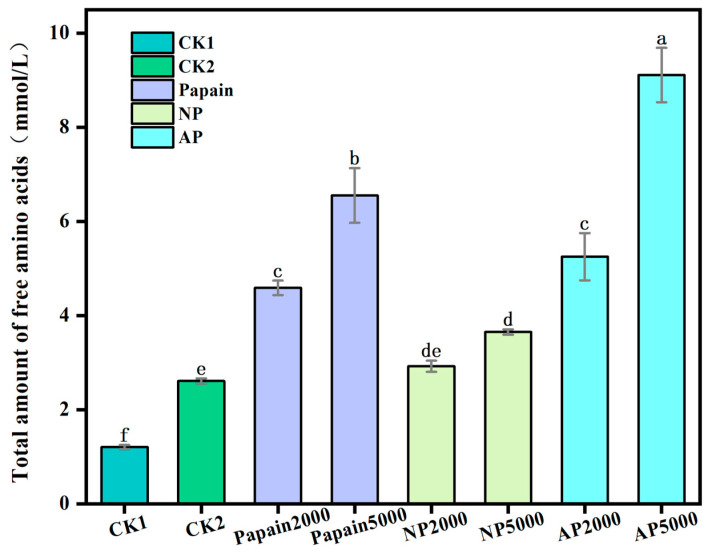
Total release of amino acids (mmol/L) in all treatment groups. CK1: crude *P. eryngii* protein powder; CK2: *P. eryngii* protein parallelly processed under pH 10 without adding proteases; Papain: papain-pretreated group; NP: neutral-protease-pretreated group; AP: alkaline-protease-pretreated group. Variance among treatment groups was compared by one-way analysis of variance (ANOVA) and post hoc Duncan Test. Groups sharing no common letter are significantly different (*p* < 0.05).

The total amino acid release of all treatment groups at digestion endpoint (I2) was analyzed by UHPLC-MS/MS (Figure 4). Compared with CK1, both the CK2 and protease pretreatment groups effectively increased the abundance of released amino acids (*p* < 0.05), with CK2 reaching values 2.16 times higher than those ofCK1 and the 5000 U dose groups of papain, neutral protease, and alkaline protease reaching values 5.43, 3.01, and 7.54 times higher than those of CK1. These results verified that both the alkaline environment and the three proteases effectively promoted the release of amino acids, where alkaline protease and papain displayed stronger potential. Comparing the doses of proteases, it was found that 5000 U of each protease was more effective than 2000 U, suggesting that an increase in protease dose enhanced the release of amino acids. Among all groups, the peak amount of free amino acids resulted from using 5000 U of alkaline protease.

**Figure 5:**
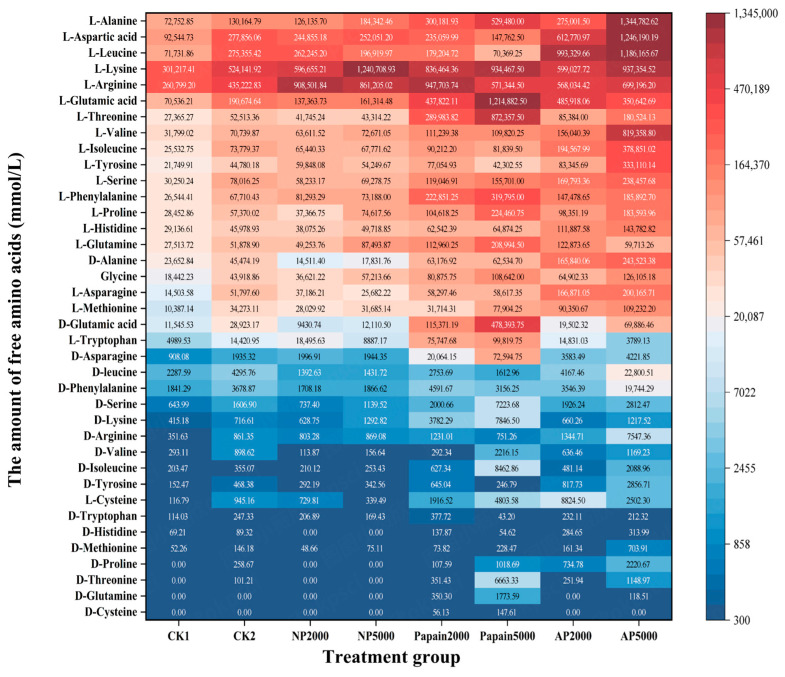
Amino acid composition in chyme at the endpoint of simulated digestion determined by UHPLC-MS/MS. CK1: crude *P. eryngii* protein powder; CK2: *P. eryngii* protein parallelly processed under pH 10 without adding proteases; NP: neutral-protease-pretreated group; Papain: papain-pretreated group; AP: alkaline-protease-pretreated group.

At digestion endpoint (I2), the amino acids released from the hydrolysis of *P. eryngii* protein mainly existed as left-handed conformers in the digestive juice (Figure 5), among which L-Alanine, L-aspartic acid, L-leucine, L-lysine, L-arginine, and L-glutamic acid were of top abundance. L-Alanine, L-aspartic acid, and L-glutamic acid can be combined with umami ingredients such as guanosine and inosine to enhance the umami taste of shiitake mushrooms [36]. Alanine showed the highest total release among all amino acids, while cysteine displayed the lowest abundance after digestion and D-cysteine was only found in the papain-pretreated groups. All indispensable amino acids required in the human diet were abundantly released as left-handed conformers, effectively supplementing the limiting amino acid (lysine) lacking in plant proteins. These results showed that *P. eryngii* protein consists of an ideal amino acid composition whose gastrointestinal release was fully increased by protease pretreatment.

After protease pretreatment on *P. eryngii* protein, there was a higher release of individual amino acids from digestion. The increased free amino acids were predominately left-handed isomers, while right-handed conformers were minimally detected. The pretreatment with papain and alkaline protease showed higher protein degradation capacity as compared with neutral protease and controls. Especially of note, the pretreatment with 5000 U of alkaline protease predominately enhanced the release of the majority of the amino acids in *P. eryngii* protein. The amino acid composition of different treatment groups varied: L-lysine and L-arginine were the most abundant amino acids in both controls as well as neutral protease group; L-lysine, L-arginine, and L-glutamic acid were the most abundant in papain group; L-leucine, L-aspartic acid, and L-lysine were most concentrated in the alkaline protease group, indicating that the cleavage sites and patterns of each protease were diverse. Compared with both controls and other treatments, the papain-pretreated group showed a notable increase in the release of D-glutamic acid, L-tryptophan, and D-asparagine. For the addition of each protease, the 5000 U dose produced more amino acids than the 2000 U, indicating that raising the concentration of protease boosted the release of amino acids.

### 3.5. Composition of Peptides Analyzed by NanoLC-MS/MS

**Figure 6:**
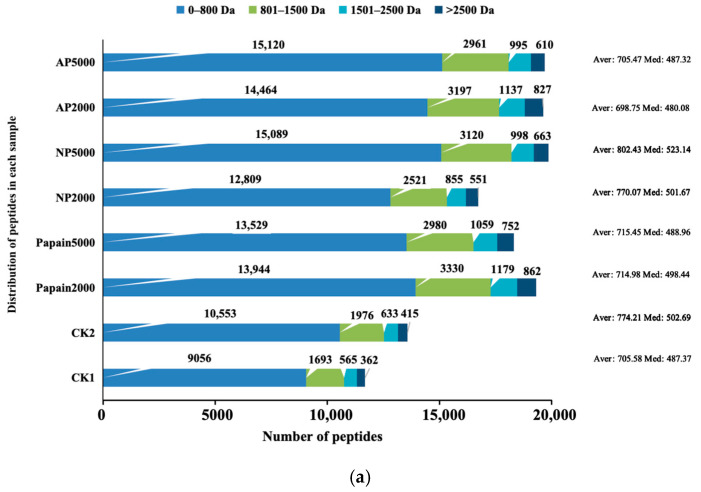

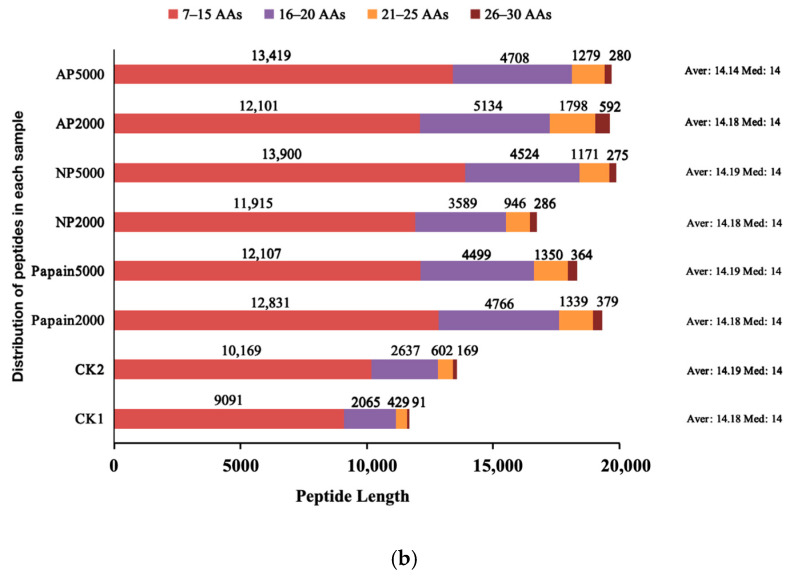
Peptide composition at the digestion endpoint determined by NanoLC-MS/MS: (**a**) histogram of peptides with different molecular weights; (**b**) histogram of peptides with different lengths. AA: amino acid; Aver: average; Med: median; CK1: crude *P. eryngii* protein powder; CK2: *P. eryngii* protein parallelly processed under pH 10 without adding proteases; Papain: papain-pretreated group; NP: neutral-protease-pretreated group; AP: alkaline-protease-pretreated group.

The distribution of molecular weight and length of peptides in the digestion endpoint products were analyzed using NanoLC-MS/MS results (Figure 6). The total number of peptides released in CK2 (13,577) and protease-treated groups (16,736–19,870) was larger than that in CK1 (11,676) (Figure 6a). Compared with CK1, CK2 released 1901 more peptides, while papain-, neutral-protease- and alkaline-protease-treated groups released 6644–7639, 5060–8194 and 7949–8010 more peptides, respectively. The peptide release in protease-pretreated groups was remarkably increased in comparison to that of both controls, especially in neutral-protease- and alkaline-protease-pretreated groups at 5000 U.

The molecular weights of peptides in controls and protease-pretreated groups were mainly concentrated below 800 Da, with peptide release numbers being 4.18–5.35 times higher than those between 801 and 1500 Da, 11.83–16.67 times higher than those between 1501 and 2500 Da, and 16.18–25.43 times higher than those over 2500 Da (Figure 6a). Peptides with smaller molecular weights typically possess higher biological activity [37], and the results indicated that protease pretreatment increased the potential for *P. eryngii* protein to release more bioactive peptides after digestion. The peptide release in the CK2 and protease treatment groups was higher than in CK1 at all of the molecular weight ranges. As for peptides with a molecular weight < 800 Da, pretreatment with 5000 U of alkaline protease or neutral protease resulted in the highest release after digestion, reaching 15,120 and 15,089, respectively. As for peptides with a molecular weight > 800 Da, the highest release was observed when pretreated with 2000 U of papain. The results demonstrated that protease pretreatment effectively promoted the release of peptides, especially small-molecule peptides, thereby improving the digestibility of *P. eryngii* and the absorbability of its hydrolyzed peptides.

Peptides were divided into four groups based on the length of their amino acid sequences to better understand the effect of protease pretreatment on the distribution of peptide lengths after in vitro simulated digestion [38,39] (Figure 6b). The small peptides composed of 7–15 amino acids were the most abundant in all samples, accounting for 61.66–77.86%. Comparing the distribution in each peptide length range across groups, small peptides consisting of 7–15 amino acids were released the most in the neutral-protease- and alkaline-protease-pretreated group at 5000 U, reaching 13,900 and 13,419, respectively. Peptides consisting of 16–20 amino acids, 21–25 amino acids, and 26–30 amino acids were found to have top release when pretreated with 2000 U of alkaline protease, accounting for 26.16%, 9.16%, and 3.02%, respectively. Protease pretreatment increased peptide yield in each length group compared with the controls. Particularly, the total peptide release from groups pretreated with 5000 U of neutral protease and alkaline protease was the most abundant. Moreover, these two groups also released larger numbers of peptides composed of 7–15 amino acids, 4809 and 4328 more than in CK1, and 3731 and 3250 more than in CK2. The more short peptides are produced during gastrointestinal digestion, the greater the bioavailability of protein that can be achieved [40]. Our results indicated that protease pretreatment was beneficial for releasing more short peptides as compared with the merely alkaline environmental control, providing reference solutions for improving the bioavailability of fungal proteins.

## 4. Discussion

The results of this study indicated that papain, neutral protease, and alkaline protease all improved the digestibility of *P. eryngii* protein. Among all groups tested, the treatment with 5000 U of alkaline protease was highlighted to show better performance in yield, particle size, chyme dispersity, amino acid and small peptide release comparing to other treatments. This study provides an efficient approach to enhance the digestion and bioaccessibility of *P. eryngii* protein, which sheds light on further exploitation of healthy mycoprotein. The speed and thoroughness of protein digestion depend on how easily digestive enzymes can access peptide bonds within the polypeptide chain [41]. It is speculated that the findings of this study are due to the action of proteases, which expose the cleavage sites of proteins to digestive enzymes, thereby accelerating the digestion process and improving digestion efficiency. Among the three tested proteases, neutral protease and alkaline protease are categorized into endonucleases. Neutral protease is a metalloprotease that selectively cleaves the peptide bond between Leu and Phe at the C-terminus [42]. Alkaline protease cleaves peptide bonds between non-terminal amino acids, such as Glu, Met, Leu, Tyr, Lys, and Gln [43]. Papain shows dual properties of endonuclease and exonuclease [44,45], which separates peptide bonds in hydrophobic regions [46]. As the cleavage site and pattern vary among different proteases, divergence was found in abundance, type and amino acid sequence of the peptide profile after gastrointestinal digestion. The results of protein particles, peptide composition, and amino acid release of our study indicated that alkaline protease showed the best cleavage effect on *P. eryngii* protein and is the most suitable for enhancing its bioavailability.

Proteases have been reported to enhance the processing performance and quality of multiple food proteins. WU et al. [23] reported that papain significantly improved anti-oxidant activity and functional properties (such as emulsifying ability, water holding capacity, solubility, and foaming ability) of soy protein, and increased in vitro digestibility as measured by nitrogen determination. LIU et al. [47] reported that alkaline protease hydrolysis after extrusion led to a positive effect on the emulsifying and structural properties of soy protein isolate, which transformed disordered structures into ordered structures, enhanced the conformational flexibility of peptides, and formed protein droplets with higher potential and smaller size. AI et al. [27] tested the effect of six proteases on egg white gel; the results indicated that the total amount of sulfhydryl group in hydrolysates, degree of hydrolysis (DH) and nitrogen solubility index (NSI) were significantly increased, while the surface hydrophobicity and emulsification decreased. Compared with other proteases, the hydrolysates formed after the flavorzyme treatment cluster with larger particle sizes. Although numerous studies have investigated the influence of enzymes on the structure and functional properties of common protein sources such as soy protein, there are few research studies that systematically analyze digestibility and bioavailability. Furthermore, limited research has delved into the effects of enzymatic hydrolysis on proteins from edible mushrooms, particularly concerning the amino acid release, hydrolysate peptide profile, and absorption mechanism. The findings of this study indicate that proteases significantly reduce the particle size and clustering of *P. eryngii* protein in gastrointestinal chyme yet have little effect on the surface potential. After protease treatment, large amounts of amino acids and peptides were released, and the proportion of small-molecular-weight peptides in the whole pool was expanded. This technical approach provides a good reference for improving the bioaccessibility of fungal proteins.

The analysis of digestibility calls for a scientifically reliable method that closely simulates the actual human digestive process. The INFOGEST in vitro model is a modular and operable standardized digestion simulation method [48] which integrates digestive enzymatic system, electrolyte environment, pH condition, and periods of gastric and intestinal phases to simulate the real digestive process in humans [49]. This model significantly reduces the required experimental costs and time, allowing for the simultaneous screening of multiple samples and nutrients in a single experiment. Compared with in vivo models, it is rapid, efficient, reproducible, and free of ethical limitations, and therefore widely applied in the study of gastrointestinal behavior of food components, pharmaceuticals, chemicals, and drugs in various research fields such as food science, nutrition, biochemistry, and pharmacology [29]. Most of the previously published studies evaluated protein hydrolysis efficiency by combing the INFOGEST model with protein quantification such as Kjeldahl method and determination kits [49,50,51]. The protein determination kits adopt different chemical reactions and staining, such as the o-pthalaldehyde (OPA) method, Bradford method and bicinchoninic acid (BCA) method. The principle of these methods involves forming colored complexes between proteins and dyes, allowing protein concentration to be calculated based on its positive correlation with the optical density (OD) value of the color [52]. However, the binding of dyes to amino acid groups and complexation by peptides is not a simple linear relationship, leading to deviations and uncertainties for quantitative analysis. In particular, the above methods are unable to effectively distinguish proteins and their degradation products in digestive mixtures, and therefore could not accurately reflect the digestion process and efficiency of proteins [53]. This study adapted and improved the methods published by COLOSMO et al. [35]. We jointly used the INFOGEST model, fluorescence microscopy technology, and LC/MS technology to effectively observe the digestion process of proteins and analyze the release and composition of amino acids and peptides at the digestion endpoint, allowing for visualized and quantified analysis of protein digestibility. Our systematic and comprehensive evaluation of the digestibility of *P. eryngii* protein lays the groundwork for further optimizing its absorption and utilization. Notably, the results of this study were obtained in a relatively closed in vitro model without simulating the mechanical forces and dynamic conditions in a real digestive system. In a dynamically open system with sustained enzyme secretion and intestinal brush nutrient emptying, the release of amino acids and peptides may be even higher [29]. Therefore, a system that mimics the biomimetic structure and gastrointestinal mobility simulation is recommended for future research. In addition, edible mushrooms contain many phytochemicals with nutritional and health benefits, such as polysaccharides, including alpha- and beta- glucans [54]. Their digestion should also be considered before moving on to applying the results to food products. ZHOU et al. [55] and XIE et al. [28] reported that polysaccharides increased the apparent shear viscosity of gastrointestinal fluids and the reduced gastric emptying rate; therefore, the binding of polysaccharides and protein in glycoproteins would impact the bioavailability of these two macronutrients. In subsequent research, the digestion mechanism of polysaccharides and their interactions with proteins in the gastrointestinal tract should be further investigated.

The composition of digestive products is a key indicator reflecting digestibility and efficiency, which determines gastrointestinal fate and subsequent absorption. XIE et al. [28] and XING et al. [56] observed that gastric digestive products from animal proteins such as beef, pork, and chicken contained significantly more peptides than those from plant-based meats. After gastrointestinal digestion, the proportion of large-molecular-weight peptides (1900–3200 Da) significantly declined, while small-molecular-weight peptides (600–1250 Da) gradually accumulated. The molecular weights of plant-based meat, pork, and beef were mainly concentrated between 800 and 1500 Da, while those of chicken were in the range of 800–1500 Da and 1500–2500 Da. Compared with meat products, protein powder and protein extract release more peptides during digestion. WANG et al. [57] found that after in vitro digestion, chicken protein powder released the most peptides (15,673), followed by beef protein (13,712) and pork protein (13,206), while soy protein only released 8353 peptides. TANG et al. [58] reported that, as detected by LC-MS/MS, a total of 8546 peptides were released from soy-protein-based infant formula after in vitro simulated gastrointestinal digestion, where small peptides consisting of 8–13 amino acids were the most abundant, accounting for 78–91% of the peptide pool after digestion. The results of this study indicated that after gastrointestinal digestion, the *P. eryngii* protein released 11,676 peptides, while protease pretreatment increased this number to reach to 16,736–19,870. These results suggested that the digestive peptide release of *P. eryngii* protein was lower than that of meat protein yet greater than that of soy protein [57]. However, after protease pretreatment, this could be improved to slightly higher levels than those of animal protein. Protease pretreatment resulted in shorter peptides after gastrointestinal digestion with smaller molecular weights, which mainly concentrated at <800 Da, consisting of 7–15 amino acids. This result is similar to the length distribution of peptides produced by soybean protein digestion reported by TANG [58]. The findings of this study indicated that protease effectively improved the gastrointestinal peptide release of *P. eryngii* protein, providing clues on its further development and application as a high-quality alternative protein.

## 5. Conclusions

*P. eryngii* is rich in high-quality protein, making it an ideal source of sustainable alternative protein. This study found that protease pretreatment improved the digestibility of *P. eryngii* protein and therefore enhanced its bioavailability. After simulated gastrointestinal digestion, the average particle size in protease-pretreated groups was significantly smaller than that in both controls, with these being more dispersed and smaller under fluorescence microscopy. The release of amino acids and peptides, especially small peptides below 800 Da, was significantly enhanced. The Zeta potential during digestion was not affected by protease pretreatment. The alkaline protease pretreatment at 5000 U was the most effective in reducing particle size, improving dispersion, and promoting amino acid release. The pretreatments with 5000 U of neutral protease and alkaline protease both showed excellent peptide release potential; the maximum release reached 1.70 times more than that of the blank control and 1.46 times more than that of the alkaline control. This study provides a technical solution for scientifically analyzing the digestibility of fungal proteins and an enzymatic approach for improving digestive efficiency, which shed light on the further development of fungal alternative proteins with high bioavailability.

## Figures and Tables

**Table 1:** Protease pretreatment conditions.

Protease	Duration min	Temperature °C	pH	Dosage U
Papain	90	40	7	1000, 2000, 3000, 4000, 5000
Neutral protease			7	
Alkaline protease			10	

## Data Availability

The original contributions presented in this study are included in the article. Further inquiries can be directed to the corresponding authors.
